# Genotype calling of triploid offspring from diploid parents

**DOI:** 10.1186/s12711-020-00534-w

**Published:** 2020-03-18

**Authors:** Kim Erik Grashei, Jørgen Ødegård, Theo H. E. Meuwissen

**Affiliations:** 1 AquaGen AS, P.O. Box 1240, 7462 Trondheim, Norway; 2 Department of Animal and Aquacultural Sciences, Norwegian University of Life Sciences, P.O. Box 5003, 1432 Ås, Norway

## Abstract

**Background:**

Polyploidy is widespread in animals and especially in plants. Different kinds of ploidies exist, for example, hexaploidy in wheat, octaploidy in strawberries, and diploidy, triploidy, tetraploidy, and pseudo-tetraploidy (partly tetraploid) in fish. Triploid offspring from diploid parents occur frequently in the wild in Atlantic salmon (*Salmo salar*) and, as with triploidy in general, the triploid individuals are sterile. Induced triploidy in Atlantic salmon is common practice to produce sterile fish. In Norwegian aquaculture, production of sterile triploid fish is an attempt by government and industry to limit genetic introgression between wild and farmed fish. However, triploid fish may have traits and properties that differ from those of diploids. Investigating the genetics behind traits in triploids has proved challenging because genotype calling of genetic markers in triploids is not supported by standard software. Our aim was to develop a method that can be used for genotype calling of genetic markers in triploid individuals.

**Results:**

Allele signals were produced for 381 triploid Atlantic salmon offspring using a 56 K Thermo Fisher GeneTitan genotyping platform. Genotypes were successfully called by applying finite normal mixture models to the (transformed) allele signals. Subsets of markers were filtered by quality control statistics for use with downstream analyses. The quality of the called genotypes was sufficient to allow for assignment of diploid parents to the triploid offspring and to discriminate between maternal and paternal parents from autosomal inheritance patterns. In addition, as the maternal inheritance in triploid offspring is identical to gynogenetic inheritance, the maternal recombination pattern for each chromosome could be mapped by using a similar approach as that used in gene-centromere mapping.

**Conclusions:**

We show that calling of dense marker genotypes for triploid individuals is feasible. The resulting genotypes can be used in parentage assignment of triploid offspring to diploid parents, to discriminate between maternal and paternal parents using autosomal inheritance patterns, and to map the maternal recombination pattern using an approach similar to gene-centromere mapping. Genotyping of triploid individuals is important both for selective breeding programs and unravelling the underlying genetics of phenotypes recorded in triploids. In principle, the developed method can be used for genotype calling of other polyploid organisms.

## Background

Polyploidy is widespread in plants and exists both in vertebrate and invertebrate animals [1, 2]. In aquaculture species, triploidy can be induced by pressure-shocking newly fertilized eggs, resulting in unreduced gametes in the females [3]. In such induced triploids, the shocking of eggs prevents the second polar body from leaving the secondary oocyte during meiosis [4]. This results in a triploid cell, in which two sets of chromosomes are inherited from the mother, and one set from the father. This practice is commonly used by the aquaculture industry to produce sterile fish for farming, and by wildlife management for stocking of sterile game fish for recreational purposes.

The Thermo Fisher GeneTitan platform is commonly used to genotype Atlantic salmon using high-density single nucleotide polymorphism (SNP) chips [5]. However, genotyping of triploids is currently not possible using the supplied Thermo Fisher software, which limits research projects for triploids of any species. The goal of this study was to develop a method for calling genotypes for triploid individuals using the output from the Thermo Fisher GeneTitan instrument. Secondary aims were to develop methods for dam- and sire-specific assignment of induced triploid offspring, and to use maternal inheritance to triploid offspring to map the maternal recombination pattern. Although the genotype calling method was tested only by using diploids and triploids, in principle, it can be extended to other ploidies as well.

## Methods

### Data

DNA was sampled from 381 triploid Atlantic salmon and genotyped on a Thermo Fisher SNP chip array with 56,177 SNPs (56 k chip). Triploidy was verified by visually identifying four distinct clusters of transformed allele strengths per individual, namely ‘contrast’ and ‘size’ (see [6]). Two individuals showed a correlation of genotypes higher than 0.99 (likely, duplicated or contaminated samples) and were removed, leaving 379 triploid individuals. In total, 3158 diploid individuals from the parent generation were also genotyped, using either the same 56 k chip or a 220,000 SNP chip (220 k chip), which had 52,458 (52 k) SNPs in common with the 56 k chip. Downstream analyses were performed using the 52 k common SNPs, or a subset of these. Two candidate parents had apparently duplicated genotypes, likely due to duplicated samples. After removal of duplicates, the number of individuals in the candidate parent dataset was equal to 3156.

In addition, 914 diploid Atlantic salmon and 116 of their previously parentage assigned parents were used to compare genotype calling methods. In total, 853 of the 914 diploid offspring had both parents assigned, i.e. trios. The 914 diploids were called using both the method developed here and using Thermo Fisher’s APT software, the Affymetrix power tools (APT) [6]. The parents were genotyped using one of the two SNP chips described above and, thus, the same 52,458 SNPs were used in all downstream analyses. Additional details are provided in the subsection ‘Calling genotypes with Affymetrix power tools’ below.

### New genotype calling method

Observations of $contrast = \log_{2} \left( {A_{signal} /B_{signal} } \right)$ (also known as ‘Delta’) were obtained for each DNA sample from the file ‘AxiomGT1.normalized-summary.txt’, which is produced by the APT software [6, 9]. The $A_{signal}$ and $B_{signal}$ are the signal strengths observed by the GeneTitan instrument for the two possible alleles (called *A* and *B*) for each SNP. Thus, the possible genotypes for a given SNP are *AA*, *AB* and *BB* for diploid and *AAA*, *AAB*, *ABB* and *BBB* for triploid individuals.

The R package “mclust” [7] was used for calling both diploid and triploid genotypes, fitting up to three and four genotype clusters, respectively, in a single dimension (the contrast). The clustering models assumed that the contrast is a mix of normally distributed variables, one for each genotype cluster, allowing for different expectations and variances for each cluster, depending on the model. The mclust package attempts to identify the underlying distributions by choosing the most likely out of two possible models for each genotype cluster. The two models are: (1) the ‘E’ model, in which each genotype cluster is assumed to have equal variances, and (2) the ‘V’ model, in which the genotype clusters can have different variances (see [7]). Not all markers will have all biologically possible clusters represented; e.g. markers of low minor allele frequency may only show the most common cluster(s). Thus, the two models are tested with the assumption that there are one, two, three, or four (for triploids only) genotype clusters in the data for a given locus. That is, for all biologically possible numbers of genotype clusters, both models (‘E’ and ‘V’) were fitted. This means that for triploid individuals, a marker could have up to four clusters (*AAA*, *AAB*, *ABB* and/or *BBB*), resulting in 4 * 2 = 8 models being tested, while for diploid individuals up to three clusters are possible (*AA*, *AB* and/or *BB*), resulting in 3 * 2 = 6 models. The integrated complete likelihood (ICL) for all models, defined as in [8] (a higher ICL is favorable), was calculated using the mclust package. ICL was chosen rather than the Bayesian information criterion (BIC) because of its tendency to favor well-separated clusters (see “Discussion” for more information). For each number of clusters $G$, the model with the highest ICL was saved, i.e. ${ \hbox{max} }\left( {ICL\left( G \right)} \right)$, where $G \in \left\{ {1,2,3,4} \right\}$ for triploids and $G \in \left\{ {1,2,3} \right\}$ for diploids. Then, the model with the highest ICL ($ICL_{1}$) was assumed to produce genotypes with the lowest genotype error rate and, thus, was chosen to classify the genotypes.

Mclust uses the iterative expectation maximization (EM) algorithm for all models, which adjusts the parameters until the most likely set of parameters is found for each model. When no starting parameter values are set, mclust uses the mean contrast of each marker as a starting point for all possible genotype clusters in the first EM-iteration. In some cases, this may result in mclust choosing a local optimum for the parameter estimates due to, e.g. uneven numbers of individuals in the different genotype classes or DNA sample bias due to differences in DNA quality (see “Discussion”). To obtain better starting values, the initial numbers of individuals in each genotype group ($n_{1} , \ldots n_{G}$, where $G$ is the chosen number of clusters) were predicted by using a rough estimate related to the SNP allele frequency (see Appendix). Then, initial clustering was done by sorting the individuals by contrast values and initially assigning the first $n_{1}$ individuals to the first (left-wise) cluster, $n_{2}$ to the second cluster, etc. The contrast means of the initial clusters were set as starting values in the EM-algorithm and used as priors for the cluster means. Further details are in Appendix.

**Fig. 1:**
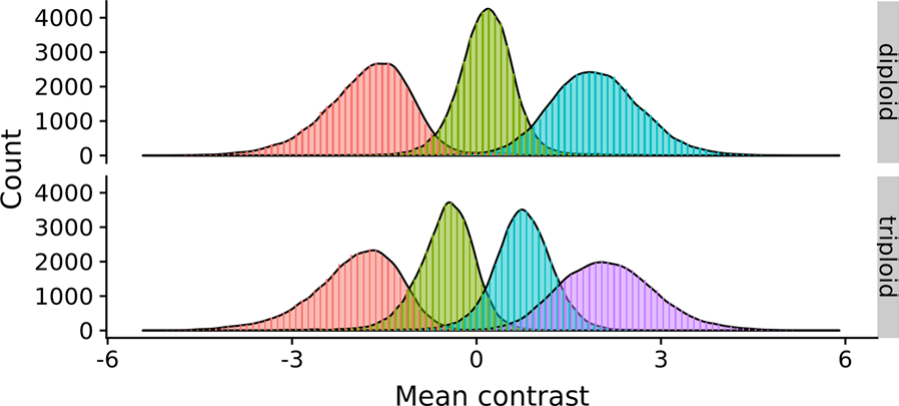
Mean contrasts across markers. Distribution of mean contrasts across all 56,177 markers with three (diploids, top panel) or four (triploids, bottom panel) predicted genotype clusters, as estimated by the expectation maximization method

In cases where all four triploid clusters are found (i.e. all genotype groups are represented for the locus in question), the lowest cluster (with respect to contrast) is assumed to correspond to genotype *BBB*, the second to genotype *ABB*, etc. The same logic applies to diploids, except that there are up to three possible clusters. If three or fewer clusters are identified for triploids, the correspondence between left-to-right cluster number and genotype value is less obvious, similar to the case of diploids having two or fewer clusters. In such cases, the genotype calls are determined by the mean contrast of each cluster. Distributions of estimated mean contrast values for all markers that are predicted to have three or four clusters for diploids and triploids, respectively, are in Fig. 1. The estimated mean of each cluster in Fig. 1 is used to call genotypes for markers with less than the maximum possible number of clusters, i.e. markers with less than four clusters for triploids and less than three clusters for diploids. These estimated reference contrast means were approximately − 1.76 (= *BB*), 0.16 (= *AB*), and 1.94 (= *AA*) for diploids, and − 1.97 (= *BBB*), − 0.50 (= *ABB*), 0.75 (= *AAB*), and 2.14 (= *AAA*) for triploids. These contrast means were estimated using the entire 56 k chip, where 44,431 and 38,792 of the 56,177 SNPs had a maximum number of clusters for diploids and triploids, respectively.

Some SNPs will not have all four (triploids) or all three (diploids) clusters because, for example, they might be fixed or have very high or very low allele frequencies. For such markers, the following approach was used: (1) retrieve the estimated mean contrast of each genotype cluster, and (2) find the closest reference contrast mean from the markers that had the maximum number of clusters (see Fig. 1) and set the genotypes to be the same as for these reference clusters. However, if two or more cluster contrast means are closest to the same reference cluster, the locus will not be used (defined as no-calls). This was the case for 950 of 11,746 SNPs in the diploid group with less than three clusters and for 4041 out of 17,385 markers in the triploid group with less than four clusters.

After choosing a model, the probabilities of belonging to each of the possible clusters are calculated for each contrast, and the cluster with the highest probability is chosen. The uncertainty is then the probability of the genotype belonging to any of the other clusters (1 minus the probability of belonging to the most likely cluster). If the uncertainty exceeds 0.15, the genotype value is defined as a no-call (i.e. a missing genotype). The threshold of 0.15 was chosen as this is the default threshold used by the APT software and, thus, provides a good comparison between the methods. Varying this threshold will result in different marker call rates, however we have not investigated the effects of varying this threshold on downstream analyses.

### Calling genotypes with Affymetrix power tools

In addition to our mclust implementation, genotypes of the 914 diploid individuals were also called by using standard Thermo Fisher APT software based on the following three-step procedure: step 1: DQC-step: generate dish quality check (DQC) values for each sample and exclude samples below a chosen threshold, step 2: call genotypes for all remaining samples and calculate sample call rates, and step 3: call genotypes again using only the individuals from step 2 with call rates above a chosen threshold (see [9] for more background and information). Thus, individuals from step 3 have higher call rates than those from step 2. On a general basis, Thermo Fisher recommends setting the DQC threshold at 0.82 and the sample call rate threshold at 97%. However, we visually inspected the curves of ordered DQC- and call rate values, and set the threshold manually. An uncertainty value (‘confidence’ in Thermo Fisher terms) is estimated by APT for each call from each sample, which is equivalent to the uncertainty calculated by mclust. The recommended and default threshold for this uncertainty is 0.15, which is what we used both for calling with APT and mclust to provide a fair comparison of the two methods (see [9] for more information). All 914 diploids had genotypes from step 3, while of the 116 known parents, 104 had genotypes from step 3 and 12 from step 2.

### Comparing APT and mclust using exclusion ratios in known trios

The 914 diploid individuals were genotyped using both APT and mclust with two goals in mind: (1) to compare the genotype calling accuracy between the two methods, and (2) to investigate if a threshold on absolute $ICL$ or $\Delta ICL$ could be used to identify high-quality markers with reduced genotyping errors, where $\Delta ICL = ICL_{1} - ICL_{2}$, i.e. $\Delta ICL$ is the difference in $ICL$ between the two most likely numbers of genotype clusters (for the best model of each cluster number). Exclusion ratios (ER) between offspring and known parents were used as the main statistic for benchmarking the methods, in addition to some other support statistics (see “Results”). Exclusions are Mendelian mismatches between offspring and parent(s), and the ER is the number of exclusions between an offspring and its parent(s) divided by the number of SNPs where the genotypes are called for both offspring and parent(s). See Tables 1 and 2 for an overview of the combinations of genotypes between trios that were regarded as exclusions when offspring are diploid and triploid, respectively. Given the sex of triploid parents, additional erroneous genotypes can be identified using the maternal-specific inheritance to triploid offspring (see Table 2).

**Table 1:** Possible Mendelian exclusions between a diploid offspring (“O exclusion”) and its diploid parents (“P1” and “P2”)

P1	P2	O exclusion	Comment
*NA*	*AA*	*BB*	One parent has NoCall
*NA*	*BB*	*AA*	One parent has NoCall
*AA*	*NA*	*BB*	One parent has NoCall
*BB*	*NA*	*AA*	One parent has NoCall
*AA*	*BB*	*AA*, *BB*	Oppositely homozygous parents
*BB*	*AA*	*AA*, *BB*	Oppositely homozygous parents
*AA*	*AA*	*AB*, *BB*	Identically homozygous parents
*BB*	*BB*	*AA*, *AB*	Identically homozygous parents
*AB*	*AA*	*BB*	One parent heterozygous and the other homozygous
*AB*	*BB*	*AA*	One parent heterozygous and the other homozygous
*AA*	*AB*	*BB*	One parent heterozygous and the other homozygous
*BB*	*AB*	*AA*	One parent heterozygous and the other homozygous

NA indicates missing genotype

**Table 2:** Possible Mendelian exclusions between a triploid offspring (“O exclusion”) and its diploid mother (“M”) and father (“F”)

M	F	O exclusion	Comment
*BB*	*NA*	*AAA*, *AAB*	Father has missing genotype and mother is homozygous
*AA*	*NA*	*ABB*, *BBB*	Father has missing genotype and mother is homozygous
*NA*	*AA*	*BBB*	Mother has missing genotype and father is homozygous
*NA*	*BB*	*AAA*	Mother has missing genotype and father is homozygous
*AA*	*BB*	*AAA*, *ABB*, *BBB*	Oppositely homozygous parents
*BB*	*AA*	*AAA*, *AAB*, *BBB*	Oppositely homozygous parents
*AA*	*AA*	*AAB*, *ABB*, *BBB*	Identically homozygous parents
*BB*	*BB*	*AAA*, *AAB*, *ABB*	Identically homozygous parents
*AA*	*AB*	*ABB*, *BBB*	Mother homozygous, father heterozygous
*BB*	*AB*	*AAA*, *AAB*	Mother homozygous, father heterozygous
*AB*	*AA*	*BBB*	Mother heterozygous, father homozygous
*AB*	*BB*	*AAA*	Mother heterozygous, father homozygous

NA indicates missing genotype

Of the 914 diploid offspring, 853 had both parents known. Thus, genotypes were called for 914 offspring, but comparisons using exclusion ratios were based on 853 known trios. For the triploid offspring, 304 known trios were used (see text regarding parentage assignment and parental sex prediction of triploids in “Methods” and “Results” sections).

### Parentage assignment of diploid parents to triploid offspring

The fraction of parents to triploids represented in the data was unknown. An exclusion-based approach was used to assign diploid parents to triploid offspring. Neither triploid nor diploid offspring can be opposite homozygotes (i.e. have exclusions) relative to their true parents through Mendelian inheritance of alleles. For example, a parent with genotype *BB* at a given SNP cannot have offspring with genotype *AA* (diploid)/*AAA* (triploid) at that SNP. However, since genotype errors can occur, a relatively small number of exclusions should be expected even in true parent–offspring pairs. The expected number of exclusions between an offspring and a non-parent individual depends on their genomic relationship, i.e. greater relatedness between individuals usually means a smaller number of exclusions. ER was used for parentage assignment instead of the number of exclusions to account for variation in individual call rates due to differences in DNA quality. Only markers for which triploid $\Delta ICL > 150$ and with parent call rates > 95% were used in the parentage assignment. ER were calculated for each pair of offspring and candidate parent. An assignment ER-threshold of 0.002 for offspring–candidate duos was applied, which means that all candidate parents with ER below this threshold for an offspring were assumed the true parents. See “Results”, for more detailed information regarding the choice of ER threshold.

### Parent sex prediction for triploid offspring

When using pressure-shock induced triploidy in fish, the offspring receives two sets of chromosomes from the mother and a single set from the father (see “Background” section). As a result, certain genotypes are not possible for a true mother of the offspring but are possible for the true father. For example, a triploid offspring with marker genotype *AAB* implies that the true mother should have at least one allele *A*, i.e. the true mother cannot have genotype *BB* at that marker. Likewise, if the offspring has a marker genotype of *ABB*, the true mother cannot have genotype *AA*. In contrast, true father and offspring can have any genotype combination, except opposing homozygotes. Using this information, true mothers and fathers can be distinguished. The “mother-specific exclusions” were used along with opposing homozygotes to construct mother exclusion ratios, coined ‘mother.ER’, which was calculated by dividing the number of mother exclusions by the number of markers for which both the offspring and the candidate mother had called genotypes. In addition, ER from non-mother-specific exclusions were also used when constructing the ‘mother.ER’ shown in our results. The same markers were used to calculate candidate ‘mother.ER’, as was used in parentage assignment (see above).

### Maternal recombinations

By pressure induced triploidy, the second polar body is not extruded during Meiosis II [4]. This implies that the sister chromatids formed during Meiosis I in the mother are still found within the ovum, along with the alleles passed down by the father, making the cell triploid. The sister chromatids passed down from the mother are identical, except for any recombinations that might have occurred during prophase I [10]. For markers for which the father is homozygous (*AA* or *BB*), the paternal allele state (i.e. a single *A* or *B*) of the offspring can be deduced, implying that maternal inheritance at that locus can also be deduced. Thus, markers for which the father is homozygous and the mother heterozygous can be used to map maternal crossovers with high accuracy given a relatively high density of such markers for multiple known offspring–mother–father trios. At the centromere, recombination is suppressed, and two identical alleles are thus inherited from the mother. On each chromosome arm, the maternally inherited alleles shift from homozygotes to heterozygotes at the location of the first crossover. A second maternal crossover (further away from the centromere) will cause the maternal alleles to shift back from heterozygous to homozygous. Hence, the triploid offspring genotypes can be used to study the recombination patterns of different chromosomes, simply by comparing genotypes of diploid parents and triploid offspring, without phasing of genotypes. This method of recombination mapping is essentially the same as gene-centromere mapping in gynogenetic diploids [11], except that induced triploids do have paternal inheritance, which is lacking in gynogenetic diploids.

In total, 304 trios of triploid offspring with assigned mothers and fathers were used to estimate maternal recombination rates. To retain the majority of the markers and still have high enough marker quality to interpret the downstream results, we chose a $\Delta ICL$ threshold of 50 and marker call rate thresholds of 0.80 for the triploid offspring and 0.95 for diploid parents. Each marker had to be mapped to a given chromosome and have at least 50 trios with informative genotypes (i.e. homozygous father and heterozygous mother). This resulted in 27,130 informative markers with a maternal recombination estimate (see Fig. 7). The markers used were placed on the ICSASG_v2 Atlantic salmon genome reference assembly [12, 13].

## Results

### Calling genotypes with mclust and APT

**Fig. 2:**
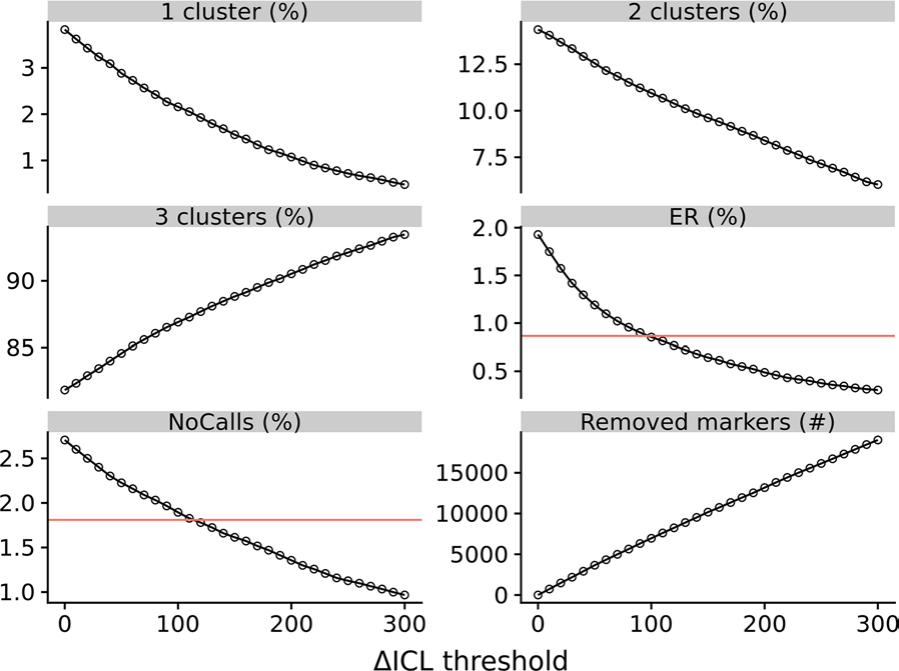
Effect of varying the $\Delta ICL$ threshold for marker selection. Statistics for diploid offspring/parent trios when varying the $\Delta ICL$ threshold for marker selection when genotypes are called by the mclust algorithm. ‘1 cluster’, ‘2 clusters’ and ‘3 clusters’ show the percentage of markers predicted to have one, two, and three clusters. ‘ER’ is the exclusion ratio shown in percent for trios. ‘NoCalls’ is the percentage of missing genotypes and ‘Removed markers’ shows the number of markers which are removed. The horizontal red lines show the values found for ‘ER’ and ‘NoCalls’ when using genotype calls from APT

Without filtering SNPs, the numbers of SNPs for diploid offspring predicted to have one, two, or three clusters were 155, 3970 and 48,333, respectively, when using APT and 2008, 7534 and 42,916, respectively, when using mclust. For each SNP called by mclust, there were two possible models: heterogenous and homogenous cluster variance. The heterogenous cluster variance model was chosen for 68% of the SNPs for diploids and for 49% of the SNPs for triploids. Using mclust, the numbers of SNPs for triploid offspring predicted to have a single, two, three, or four clusters were 3149, 3528, 10,708, and 38,792, respectively. Of the 9542 SNPs with less than three clusters called by mclust for diploids and that were used in downstream analyses, 724 were given 100% no-calls, due to insufficient separation of clusters (see subsection “New genotype calling method” in “Methods”). For triploids, there were 3620 such markers. Figure 2 shows indicator statistics of mclust marker calling quality in diploids for different thresholds of $\Delta ICL$. Increasing $\Delta ICL$ resulted in a lower ratio of SNPs with one or two clusters for diploids, while the ratio of SNPs with three clusters increased. Furthermore, the ratio of Mendelian errors (ER) decreased as $\Delta ICL$ increased, indicating that increasing the threshold for $\Delta ICL$ improves calling quality. This was supported by the decreasing ratio of missing genotypes (‘NoCalls’), which indicates that higher thresholds for $\Delta ICL$ results in retaining SNPs that have good separation of genotype clusters, i.e. SNPs with a low call uncertainty. The red horizontal lines in Fig. 2 show the values achieved by using the genotype calls from APT with all SNPs included in the analyses. APT achieved fewer Mendelian errors (ER) and fewer missing genotypes (‘NoCalls’) than our mclust implementation when all SNPs were used. Mclust needs a $\Delta ICL$ threshold of ~ 110 to obtain similar levels of ER and no-calls as APT, which resulted in the use of ~ 7500 fewer SNPs.

**Fig. 3:**
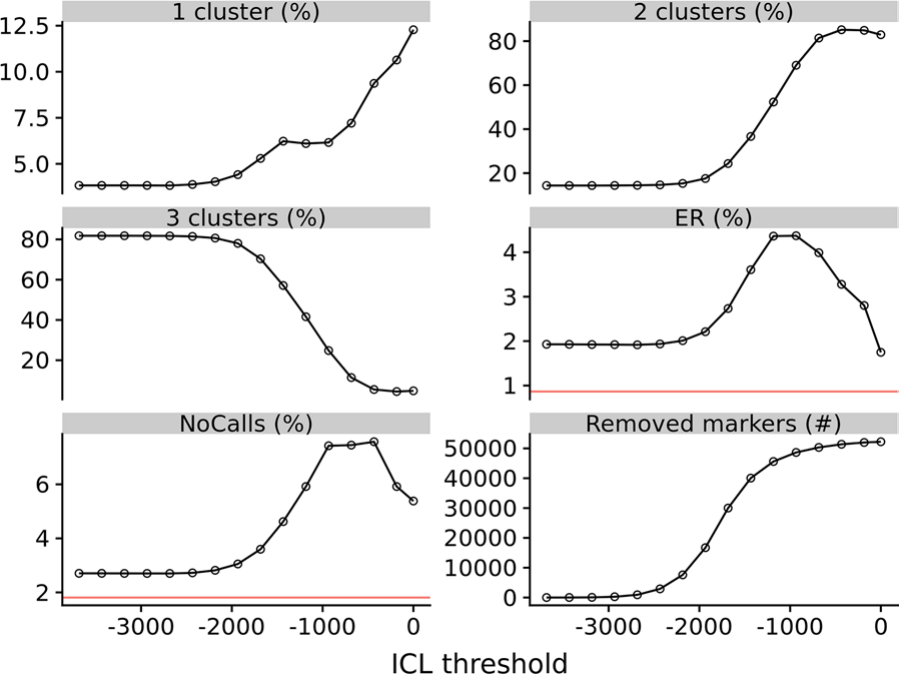
Effect of varying the $ICL$ threshold for marker selection. Statistics for diploid offspring/parent trios when varying the $ICL$ threshold for marker selection when genotypes are called by the mclust algorithm. ‘1 cluster’, ‘2 clusters’ and ‘3 clusters’ show the percentage of markers predicted to have one, two and three clusters. ‘ER’ is the exclusion ratio shown in percent for trios. ‘NoCalls’ is the percentage of missing genotypes and ‘Removed markers’ shows the number of markers which are removed. The horizontal red lines show the values found for ‘ER’ and ‘NoCalls’ when using genotype calls from APT

Figure 3 shows the same statistics as Fig. 2 after removing SNPs below an $ICL$ threshold (note: not $\Delta ICL$), i.e. a threshold on the $ICL$ of the most likely model. All investigated $ICL$ thresholds result in higher ER and NoCalls than was achieved by APT without SNP quality filtering. Because the variability of ER and no-calls seemed erratic, we decided not to use $ICL$ as a marker quality filtering statistic in downstream analyses.

**Fig. 4:**
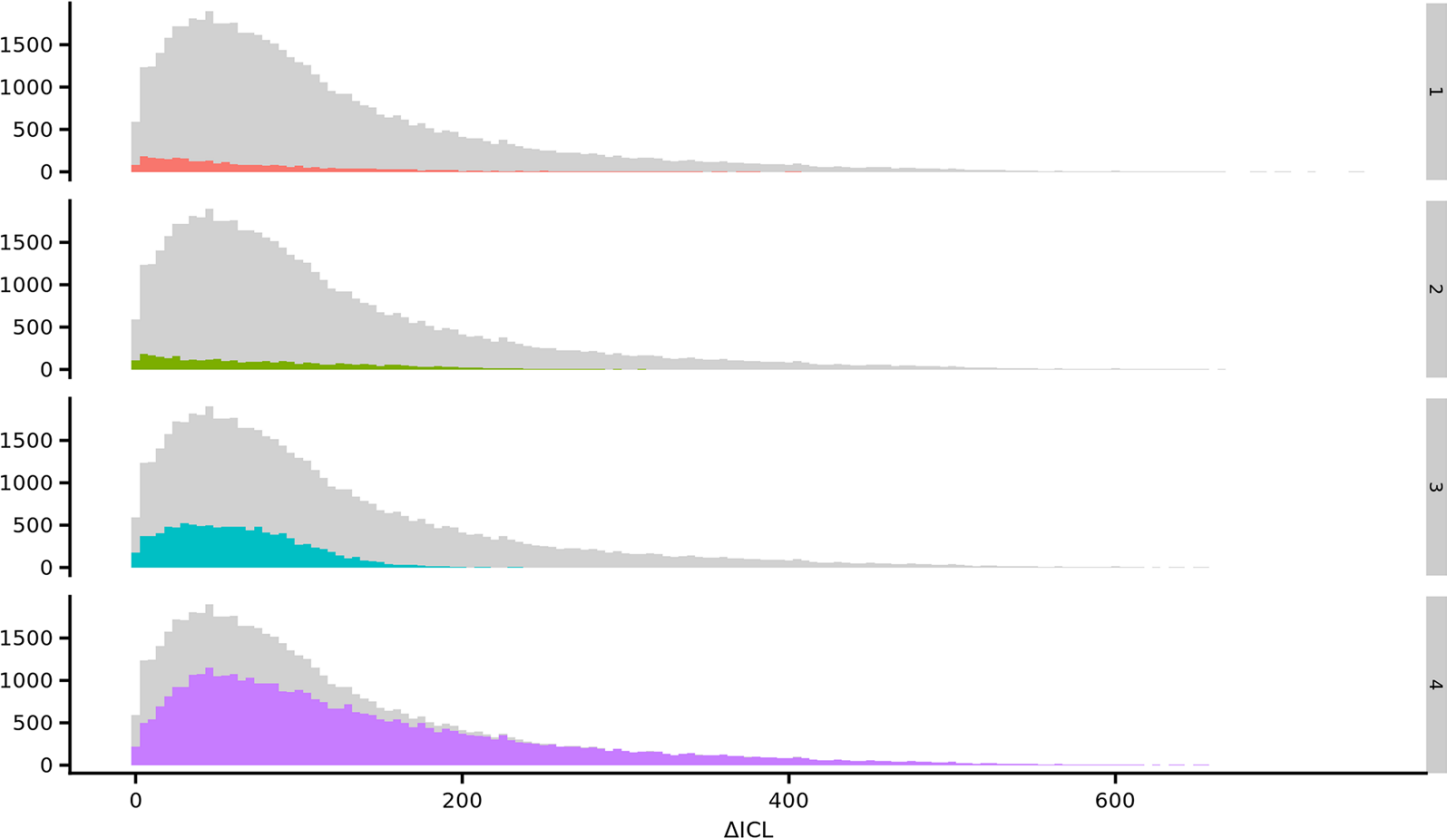
$\Delta ICL$ distributions for markers genotypes in triploids. Distribution of $\Delta ICL$ values for markers predicted to have one (top panel) to four (bottom panel) genotype clusters in triploids. Markers with red, green, cyan and purple indicate 1, 2, 3 and 4 predicted genotype clusters, respectively, while the gray bars shows the total number of markers for each value of $\Delta {\text{ICL}}$

The histograms in Fig. 4 show that the $\Delta ICL$ achieved for one, two, and three clusters were roughly the same in triploids, while higher $\Delta ICL$ could be achieved for four clusters.

### Parentage assignment of diploid parents to triploid offspring

**Fig. 5:**
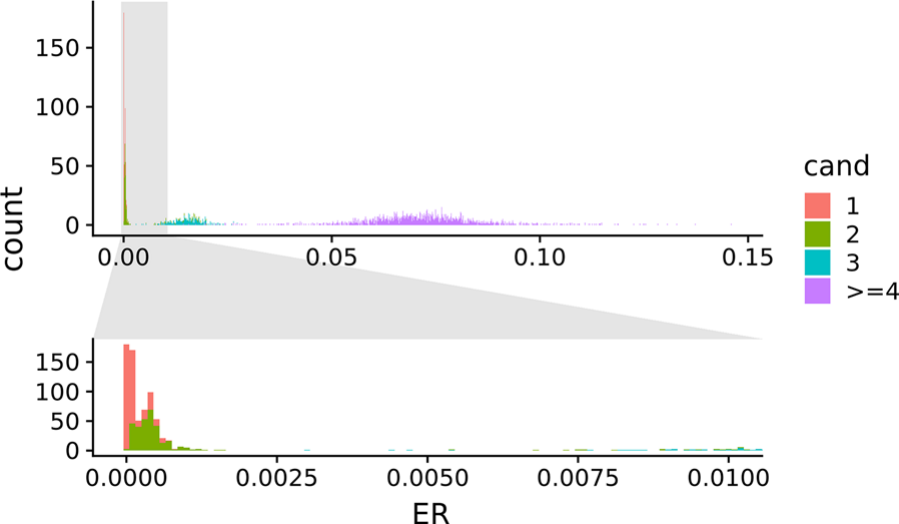
Triploid exclusion ratios. Exclusion ratios (ER) for duos of triploid offspring and diploid parents are shown for the duos with lowest ER (red), second-lowest ER (green), third-lowest ER (turquoise) and 2000 randomly sampled ER from fourth lowest and above (purple)

The QC filtering of SNPs for parentage assignment based on triploid $\Delta ICL > 150$ and parent call rates > 95% resulted in retaining 35, 375, 238, and 13,258 SNPs with one, two, three, and four genotype clusters, respectively, which resulted in the use of 13,906 SNPs for this parentage assignment. The ER between offspring and their first, second, third, and less likely candidate parents are shown in the top panel of Fig. 5, while the bottom panel zooms in on the best fitting parent candidates. The lowest ER between all triploid offspring and their third most likely candidate parent (i.e. the closest-fitting non-parent) was ~ 0.003. Consequently, a 0.002 assignment threshold for offspring–candidate duos was applied, which also fitted well, based on visual inspection of Fig. 5. In other words, the candidate parent in any duo with an ER < 0.002 was assigned and assumed to be a true parent. At least one parent was assigned to all 379 triploids, and 304 were assigned both parents. Lacking assignments were likely due to genotypes of some parents being absent in the dataset.

### Parent sex prediction for triploid offspring

**Fig. 6:**
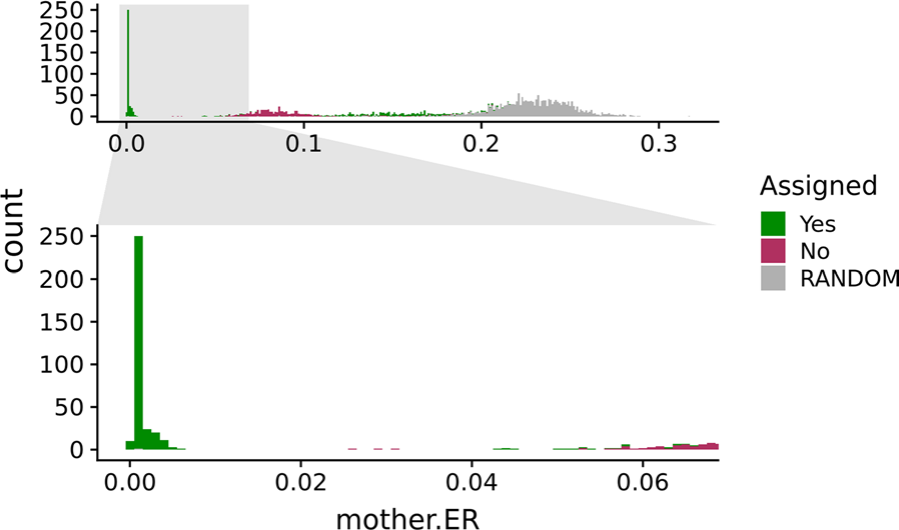
Triploid maternal exclusion ratios. Maternal exclusion ratios (mother.ER) for duos of triploid offspring and diploid candidate mothers are shown for the assigned (green) and unassigned (maroon) duos and 2000 generated ‘mother.ER’ values from randomly sampled parent candidates, i.e. both sampling males and females which can possibly be true mothers (grey)

A similar procedure as for parentage assignment was used for assignment of mothers of triploid offspring, using the mother exclusion ratios (‘mother.ER’). Figure 6 shows the ‘mother.ER’ between assigned, unassigned, and random pairs of offspring–parent candidates (note that ‘Assigned’ in Fig. 6 is for parentage assignment, not mother assignment). The minimum ‘mother.ER’ of the third-best parental candidates was ~ 0.022, thus we set the ‘mother.ER’ assignment threshold at 0.02. Any assigned parent with a ‘mother.ER’ < 0.02 was assigned as mother, and any assigned parent with ‘mother.ER’ ≥ 0.02 was assumed to be the true father. This resulted in 58 assigned mothers and 65 assigned fathers. No mothers were assigned as fathers, or vice versa. In total, 304 offspring were assigned both their mother and father (the same as the two parents assigned above), 14 were assigned a mother only, and 61 were assigned a father only (i.e. as above, all individuals were assigned a father, a mother, or both). No offspring were assigned two apparent mothers or two apparent fathers.

### Investigating different thresholds for $\Delta ICL$ and marker call rate

In addition to the $\Delta ICL$ threshold statistic explored above, marker call rate is another marker quality statistic that is often employed when analyzing genotype datasets. Marker call rate is related to ICL, as both call rate and ICL use call uncertainty as a measure of marker quality. $\Delta ICL$ provides a probabilistic penalization of the mixture model likelihood [8], whereas marker call rate is the fraction of genotypes that fall below a pre-defined uncertainty threshold. We chose the uncertainty threshold of 0.15, i.e. all genotype calls above this threshold were defined as no-calls (missing genotypes) for both triploid offspring and diploid parents. Table 3 shows that increasing either the marker call rate threshold or the $\Delta ICL$ threshold tended to decrease Mendelian errors, i.e. decrease the ER, but also increased the number of removed markers. Note that, for the parents, we always used a marker call rate threshold of 95%.

**Table 3:** Marker quality filtering using different thresholds for call rate and $\Delta ICL$

$\Delta ICL$ threshold	Marker call rate threshold				
	0.80	0.85	0.90	0.95	1.00
*0*	0.02604 (3668)	0.02573 (3774)	0.02505 (4505)	0.02338 (7411)	0.0263 (29,905)
*50*	0.01582 (16,329)	0.01571 (16,340)	0.01545 (16,423)	0.01471 (17,526)	0.01461 (34,111)
*100*	0.00924 (29,888)	0.00916 (29,892)	0.00906 (29,902)	0.0089 (30,076)	0.00723 (39,535)
*150*	0.00433 (38,521)	0.00433 (38,521)	0.00433 (38,521)	0.0043 (38,535)	0.00287 (43,563)
*200*	0.00206 (43,557)	0.00206 (43,557)	0.00206 (43,557)	0.002 (43,561)	0.00114 (46,168)
*250*	0.0007 (46,679)	0.0007 (46,679)	0.0007 (46,679)	0.0007 (46,679)	0.00051 (47,936)
*300*	0.00037 (48,705)	0.00037 (48,705)	0.00037 (48,705)	0.00037 (48,705)	0.00029 (49,286)

Filtering markers using trios of triploid offspring and diploid parents with predicted sexes using marker call rate thresholds (top row in bold) and thresholds for $\Delta {\text{ICL}}$ (left column in italic). The first number in each internal cell is the overall ER for all markers and all trios where there are informative genotypes (i.e. trios where offspring and at least one parent has called genotypes, see Table 2). The number of removed markers is shown in parenthesis.

### Maternal recombination rates

**Fig. 7:**
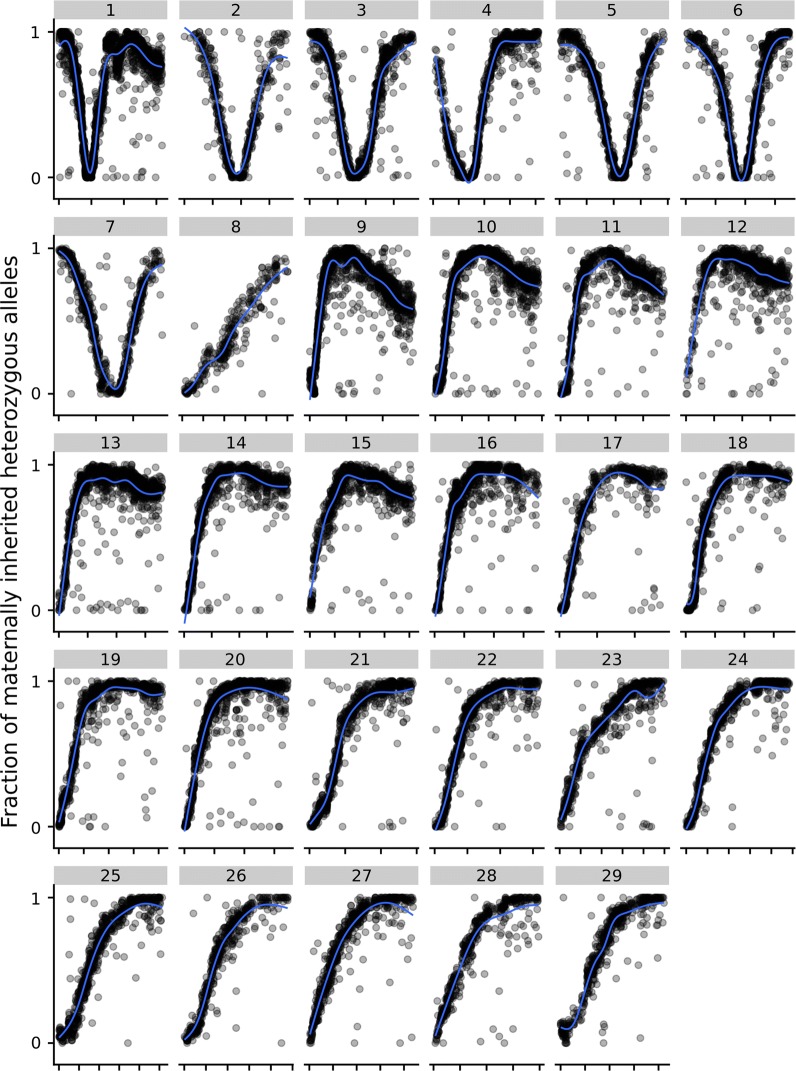
Triploid maternal recombination events. The fraction of heterozygous (*A* and *B*) alleles inherited from mothers for informative loci along each of the 29 chromosomes in Atlantic salmon. The x-axis is marker position on each chromosome and is scaled by chromosome size. All markers are required to have at least 50 trios with informative genotypes

Figure 7 shows the estimated maternal recombination fraction along each of the 29 chromosomes in the Atlantic salmon genome by looking at where the triploid offspring inherited the homozygous (*AA*/*BB*) or heterozygous (*AB*) allele from the mother (see “Methods”). The region with the lowest maternal recombination fraction on each chromosome was at the centromere, where recombination is known to be suppressed. In [13], chromosome 8 was reported to be metacentric, but in Fig. 7 it appears as acrocentric or telocentric. However, the p-arm of chromosome 8 contains highly repetitive regions and, therefore, few or no markers from this region may be represented on the SNP chip (personal communication with S Lien, see also [14]).

## Discussion

Sterile triploid Atlantic salmon have been produced for decades and differences in traits between the triploid and diploid Atlantic salmon have been observed [15]. To assign parentage, to identify population background, or to perform any kind of genetic analysis of triploids with genotype data requires methods for genotype calling in triploid individuals.

Calling SNP genotypes using sequencing data relies on the number of alleles that is called at a certain locus, and to know how this pattern varies for the two homozygous and the different heterozygous genotype groups [16, 17]. Conceptually, this differs from calling genotypes based on the aggregated light signal created by the Thermo Fisher GeneTitan instrument, as investigated here, because each allele has already been called in the sequence data. To the best of our knowledge, no official software for calling triploid genotypes using output from the Thermo Fisher GeneTitan instrument currently exists. However, software for polyploid genotyping has been created by other groups, such as the R package fitPoly [18, 19]. We chose to use mclust due to our familiarity with its functionality and its substantial documentation, frequent updates, and extensive use. Both mclust and fitPoly use mixture models and the EM algorithm to estimate parameters. Thus, fitPoly and mclust implementations may give similar results but significant differences cannot be ruled out. See “Calling genotypes with mclust and APT” subsection below for more discussion on this. However, a comparison between mclust and fitPoly was outside the scope of this study. In [20], Serang et al. use graphical Bayesian modelling to incorporate information on population allele frequencies or parental genotypes into the model to achieve increased genotype calling accuracy. Although we did not use information on allele frequencies or parental genotypes, this information could be used to increase the accuracy of the genotype calling, as shown in [19]. However, we estimated starting values for the EM algorithm and priors for the cluster means based on a rough estimate of allele frequencies (see Appendix). Another approach could be to correct genotypes by using allele frequency information after genotype calling has been performed. Unlike in [21], offspring and parents were not called using the same method in our dataset. If any bias in the calling procedures used by APT or mclust exists, it can lead to incorrectly assuming that there are fewer Mendelian inconsistencies when calling genotypes using APT in both parents and offspring, as opposed to calling parents with APT and triploid offspring with mclust. We have not investigated whether this is the case in our dataset. Although we focused on Atlantic salmon, the method can be extended to other polyploid species, e.g. in plants [1].

### Calling genotypes with mclust and APT

Genotypes were called in both triploid and diploid offspring through Gaussian finite mixture modelling using the EM algorithm, implemented in the R package mclust. The R packages fitTetra 1.0 [19] and fitTetra 2.0 [21] were developed by the same group that developed the fitPoly package [18, 19], and they all use the same underlying EM-based algorithm for genotype calling. However, fitTetra 1.0/2.0 are limited to calling tetraploid genotypes only. Both fitTetra 1.0/2.0 [19, 21] and fitPoly [18, 19] assume a common variance for all genotype clusters for a marker by transforming the intensity signal ratios to obtain approximately constant variance. Our implementation runs two models for each marker, one model that assumes equal variance and another model that allows for heterogeneous variance of the clusters, where the model with the highest ICL value is assumed to be the best. With our implementation, it seems that both models are useful, as 68 and 49% of the markers had heterogenous cluster variance for diploids and triploids, respectively. The reason for the difference between rate of markers with heterogenous cluster variance between diploids and triploids is unknown, but it can be hypothesized that the increased overlap of clusters in triploids causes mclust to prefer the heterogenous cluster variance model. Both fitTetra 1.0 and 2.0 use the Bayesian information criterion (BIC) to score models. However, genotype clusters are well separated for markers with high quality, which harmonizes well with the ability of ICL to penalize models with a low degree of cluster separation [8]. Variation in DNA quality in a sample dataset can affect the signal intensities from DNA hybridizing with the probes on the SNP chip [22–24]. This can result in clusters being distributed non-Gaussian. The algorithm can fit additional Gaussian clusters to account for violations of model assumptions [25]. Hence, the number of fitted clusters can exceed the number of biological genotype groups. Biernacki et al. [8] showed that BIC tends to overestimate the number of clusters when the model fits the data poorly. Since genotype clusters are expected to have different contrast means (i.e. separated clusters), and since factors such as heterogeneous sample quality can result in model assumptions to be violated, we chose ICL over BIC as a model selection criterion.

All genotype calls were given an estimate of uncertainty based on the probability of the genotype belonging to another cluster than that with the highest probability. The threshold for this was set to 0.15, which means that all genotypes with an uncertainty > 0.15 were no-calls (i.e. missing genotypes). Decreasing the threshold for uncertainty would decrease the call rate of each marker and, thus, the number of markers used in downstream analyses. In our opinion, results from the downstream analysis indicate that the genotype calling method was appropriate and gave reliable and trustworthy results.

Salmonids have been through several genome duplication events and their genomes are in a state of re-diploidization from the last genome duplication event, which resulted in a tetraploid genome [13]. That is, different regions of the Atlantic salmon genome are still in a tetraploid state. When creating SNP chips for such a genome, it is necessary to ensure that the SNP is in a region of the genome that is not duplicated, or that the SNP is in a tetraploid region where only one of the homologues is polymorphic for the SNP (“semi-fixed”). Having markers targeting “semi-fixed” SNPs can complicate the calling procedure because shifts in contrasts can be observed. For example, for a “semi-fixed” SNP with possible genotypes *AAAA*/*AAAB*/*AABB*, the contrast for the marker targeting this SNP can be shifted towards the right (i.e. towards the ‘*A*’-allele). Furthermore, hybridization affinity between the probes on the SNP chip may not be equal for the *A*- and *B*-alleles, which can also result in shifts of contrasts for the genotype. The problem of duplicated regions and/or differences in allele affinity for hybridizing with the probe is expected to be worse in triploids. Tetraploid regions in diploids become hexaploid regions in triploids, potentially resulting in more severe shifts in allele affinity compared to normal diploids (or tetraploids). In addition, since triploids have two heterozygote groups, there is an elevated risk of overlap between the clusters (see Fig. 1). We did not investigate to what degree any of the SNPs on our chip were affected by such semi-fixed SNPs.

APT uses the BRLMM-P algorithm, which was developed by Affymetrix (now Thermo Fisher) [26]. Many elements of the BRLMM-P algorithm are similar to the current implementation of mclust, e.g. estimation of contrast cluster means and variances and use of priors. However, one key aspect that differentiates APT from the mclust implementation is the use of covariances between different cluster means [26]. Currently, software limitations prevent this from being implemented with mclust. Another difference is that APT provides uncertainty estimates for each genotype call from markers that have only one genotype class (e.g. monomorphic markers within dataset), while mclust does not. Thus, no-calls are produced for such markers by APT but not by mclust. Although possible, we did not investigate implementing this in mclust. This could account for some of the increase in ER when the $\Delta ICL$ threshold is low, since the fraction of markers with one cluster was increased (Fig. 2).

Figure 1 shows the distribution of estimated mean contrasts in each of the four genotype clusters for markers with three/four (for diploids/triploids) predicted clusters. Note that these distributions are across all markers (containing many different marker clusters skewed in different directions), and therefore there is more overlap between clusters than would be the case for individual markers. There was also some evidence for a widening of the contrast space from diploids to triploids, i.e. the contrast cluster means ranged from − 1.76 (= *BB*) to 1.94 (= *AA*) for diploids and from − 1.97 (= *BBB*) to 2.14 (= *AAA*) in triploids. All DNA extractions were normalized to the same concentration, so the reason for this difference in range is not known. However, normalization of DNA concentration is not only based on the initial concentration of DNA, but also on the concentration of other components such as protein, which may result in a higher final DNA concentration for the triploids. In any case, the contrast means, as expected, overlapped more in triploids than in diploids because of the two heterozygote clusters, making it more difficult to distinguish between clusters.

Figure 4 shows the distribution of $\Delta ICL$ for markers with one to four predicted clusters for triploid individuals. The markers that had four predicted clusters seemed to be able to achieve higher $\Delta ICL$ than what was possible with fewer predicted clusters. Markers with three or less biological clusters are expected to have low minor allele frequencies (MAF). For such markers, the number of observations within some of the clusters is likely very small, making estimates of cluster parameters less precise, and thus limiting ICL due to uncertain clustering.

APT uses pre-determined priors for means and variances, with equal priors for all markers as default. These priors were also used in the current study.

A small fraction of the induced triploids is expected to have failed triploidization. If any of the individuals assumed to be triploid are in fact diploid, calling accuracy is expected to decrease. However, the presence of diploids in the triploid dataset was deemed unlikely in this study, as inspection of (transformed) allele strength distributions revealed four distinct clusters for all individuals that were assumed to be triploids (see “Methods”).

### Comparing APT and mclust using exclusion ratios in known and assigned offspring–parent configurations

To compare our mclust implementation with APT, we called the same diploid offspring with both mclust and APT. In Fig. 2, it is clear that, without marker quality filtering, APT achieved lower Mendelian error rates compared to our implementation of mclust. When markers were filtered based on $\Delta ICL$, around 7 to 8000 markers had to be removed before Mendelian error rates achieved with mclust and APT were comparable. Figure 2 also shows that, without marker quality filtering, the percentage of missing genotypes (no-calls) was higher for mclust than for APT.

Knowing the parents’ sex enabled us to identify more Mendelian exclusions for triploid offspring (see Table 2) than for diploid offspring (see Table 1). As a result, exclusion rates for diploid and triploid offspring could not be directly compared. Higher error rates are expected in triploids due to more overlapping genotype clusters (e.g. Fig. 1). Because of this, the genotype error rate (or e.g. ER) should be estimated separately for triploids.

We used APT to call the parents of both triploid and diploid offspring. Consequently, there may exist bias in favor of APT. Hence, mclust may appear to give more mismatches than APT between genotypes of offspring and parents (always called with APT), which would affect the estimated Mendelian error rate (ER) for both diploid- and triploid offspring.

### Parentage assignment of diploid parents to triploid offspring

A threshold of $\Delta ICL > 150$ was chosen to retain high-quality markers for parentage assignment of diploid parents to triploid offspring. This arbitrarily large number was chosen to ensure that accurate parentage assignment was used in downstream analyses. Note that the threshold of $\Delta ICL > 150$ was only used for parentage assignment of the triploids, not in downstream analyses, where other thresholds were investigated and chosen. The fact that parentage could be assigned to a substantial number of triploid offspring with clear differences in exclusion rates for assigned parents compared to non-assigned parents is an indication that the calling of triploid genotypes was successful. Parent sex prediction, comparisons between our implementation and APT, and mapping of maternal recombinations, all depend on correct parentage assignment. This is another indication that both the triploid genotyping and parentage assignment were successful and accurate. Applying an ER-threshold of 0.002 worked well in this dataset but may not be applicable in all situations (it may depend on, e.g. the SNP chip, genotype errors, or relatedness between individuals in the sample). The ER-threshold should be set lower than the minimum ER of the third most likely candidate for all duos (assuming that duplicates or clones of parental DNA are not present in the data). Furthermore, (visual) inspection of the ER distribution is required to locate the probable region of true parental ER’s. Parentage assignment using high-density SNP genotypes and exclusions (opposing homozygotes) is frequently used for parentage assignment of diploid offspring (e.g. [27–29]). Parentage assignment in triploid Pacific oyster (*Crassostrea gigas*) offspring with diploid mothers and autotetraploid fathers was performed by Miller et al. [30] using microsatellite markers. Nonetheless, we are not aware of any case where triploid offspring have been assigned diploid parents using high-density SNP data.

### Parent sex prediction

Accurately identifying sex in salmonids using genotypes is not trivial [31, 32]. In pressure-induced triploids, the fact that mothers contribute two alleles to their offspring and fathers one allele can be used to separate the already assigned parents into mothers and fathers. Two assigned “mothers” or “fathers” indicate a false assignment, either by incorrectly assuming triploidy in diploid offspring or by duplicated parental samples. In our analysis, all assigned parents were consistently assigned as either fathers or mothers across all triploid offspring.

Since the parent candidate dataset included closely-related individuals, several candidates were likely closely related with the true parents. Close relatives of the mother will have a high fraction of genotypes that resemble the genotypes of the true mother, which gives such candidates relatively low ‘mother.ER’, even compared with the true father (Fig. 6).

In the ER-based parentage assignment (Fig. 5), sex of the parent was not considered. Still, we observed some differences in ER between the sexes, with lower average ER for mother–offspring pairs. This may be explained by the fact that the mother contributes two alleles and the father one allele. For example, if the true mother has genotype *AA*, the triploid offspring can have genotypes *AAA* and *AAB*. In contrast, a true *AA* father can have triploid offspring with genotypes *AAA*, *AAB* and *ABB*. The latter genotype is more likely to be misinterpreted as *BBB* (see Fig. 1), generating a false exclusion genotype.

### Maternal recombinations

Figure 7 shows an increase in maternal recombination rates when moving away from the predicted centromeric region for all 29 chromosomes [14]. By visual inspection, the centromeric regions for the most part aligned well with what was reported by Lien et al. [14]. However, chromosome 8 was reported to be metacentric in [14], while we observed it to be acrocentric or telocentric probably due to a lack of markers on the p-arm of chromosome 8, see “Results”. Figure 7 shows that inheritance of maternal alleles was highly dependent on the distance between the locus and the centromere. For loci that are heterozygous in the mother and close to the centromere, the offspring usually inherited two identical maternal alleles, while for loci far from the centromere the offspring usually inherited two different maternal alleles. Thus, the inherited alleles are not expected to be in Hardy–Weinberg equilibrium. Estimating the number and position of recombinations is possible for each individual mother by searching for transitions from homozygous to heterozygous maternal inheritance.

Figure 7 shows the fraction of offspring that inherited heterozygous alleles from the mother at different positions along each chromosome (only informative genotypes were included, i.e. heterozygous mother and homozygous father). There were signs of interference for all chromosomes in Fig. 7. Under a model of no interference, secondary recombinations on the chromosome arms would frequently occur. Instead, all chromosomes showed a rapid increase in the fraction of heterozygous maternal alleles when moving away from the centromere, with little indication of secondary recombination (which would result in homozygous inheritance of maternal alleles). For some of the bigger acrocentric chromosomes, the maternally inherited heterozygous fraction approached 1 before it started to decline. This was most prominent for chromosome 9 and might suggest that interference was affected by distance from the last recombination event. Since induction of triploidy by pressurization occurs after prophase, the pattern of recombination should not be different when ordinary oocytes are formed for haploid inheritance of alleles.

Because this study focused on genotyping polyploids, and specifically triploids, further investigations on the implications of maternal recombinations were deemed outside the scope of the current study.

### Application to other methods for creating triploid offspring

Other ways of producing triploid individuals are possible, such as mating tetraploids with diploids [33, 34]. In such cases, the methods used here for genotype calling and ER-based parentage assignment can still be used (given that genotypes can be called for the tetraploid parent), but the methods used here for parent sex detection and mapping of recombination events are not necessarily applicable.

## Conclusions

We have developed a technique for genotyping triploid individuals using allele signals from the Thermo Fisher GeneTitan genotyping platform, or other platforms that use light intensity for estimating the allele hybridization ratio. Using the called triploid genotypes, diploid parents could be assigned to induced triploid offspring and sex of the assigned parents could be predicted. No a priori information about the parents was needed, except their genotype information (not including any sex-linked markers). Furthermore, the genotypes of triploid offspring and their assigned parents were used to map maternal recombination events along the chromosomes. The methods and results of this study can be used for further genetic analyses (genomic prediction, genome-wide association studies) of phenotypic traits recorded in triploids as well as their genetic covariance with phenotypic traits recorded in diploids.

## Data Availability

The data that support the findings of this study are available from AquaGen AS but restrictions apply to the availability of these data, which were used under license for the current study, and thus are not publicly available. Data are, however, available from the authors upon reasonable request and with permission of AquaGen AS.

## Appendix

### Providing the mclust package informative starting proportions and prior mean parameter estimates

In the first iteration of the expectation maximization (EM) procedure, the overall mean for each variable is normally used as starting parameters for $\mu_{k}$ by mclust [7]. In our experience, this may lead to incorrect classifications, and informative starting parameters and priors can be used to increase classification accuracy. As with APT, the contrast variable was deemed the most informative with respect to genotype classification, and informative starting values and prior parameters were thus estimated for this variable.

Assuming that DNA is sampled from random individuals in a population, the number and size of clusters depend on the allele frequency of the marker. For example, if the allele frequency of allele *A* is 50%, in diploids, we would expect to see equally-sized clusters for the two opposing homozygotes (*AA* and *BB*) and a heterozygote *AB* cluster with twice the size of either the *AA*- or the *BB*-cluster. If the minor allele frequency is low, some genotype classes may be absent from the data, leading to a reduced number of clusters. As discussed in the main text, the maternal inheritance of alleles for induced triploid offspring depends on the maternal recombination rate. This may result in genotype groups not strictly adhering to Hardy–Weinberg equilibrium. However, it should still be better to have slightly imprecise starting estimates and priors compared to using the overall contrast mean as the starting point for all genotype clusters.

When estimating informative priors and starting parameters for mclust for a defined number of possible cluster classes (e.g. $G \in \left\{ {1,2,3,4} \right\}$ in triploids), the number of individuals in cluster class C (i.e. having genotype C) approximately follows a binomial distribution:

1 $$C\sim bin\left( {G - 1,p} \right),$$

where $p$ is the success probability (affected by the allele frequency, but not necessarily equivalent to it). A priori, the $p$ parameter is unknown, but can be roughly estimated from normalized contrast values $\left( {d_{norm} } \right)$:

2 $$d_{norm} = \frac{{d - d_{min} }}{{d_{max} - d_{min} }},$$

3 $$\hat{p} = mean\left( {d_{norm} } \right),$$

where $d$ is the contrast value for the marker obtained from the Thermo Fisher genotyping platform for an individual, $d_{max}$ is the maximum and $d_{min}$ is the minimum contrast values for the marker. Each SNP on the chip is a collection of probes. The light signal produced when the different alleles are hybridized with probes for a single marker on the SNP chip can reach maximum intensity when 100% of the probes are hybridized. However, it is not certain that 100% of the probes hybridize with alleles, and thus the contrast range may vary. By transforming the contrast range such that the normalized contrasts are in the range between 0 and 1, we are able to use $\hat{p}$ as the success parameter in a binomial distribution. From this, we can roughly estimate the number of genotypes in each genotype class as:

4 $$\hat{n}_{c} = Pr\left( {C = c|G - 1,\hat{p}} \right) \cdot n,$$

where $n$ is total number of individuals genotyped for this marker and $Pr\left( {C = c|G - 1,\hat{p}} \right)$ is the binomial probability of observing $c$ successes in $G - 1$ trials with success probability $\hat{p}$.

For the marker in question, contrast values were then sorted from smallest to largest, and the first $\hat{n}_{0}$ observations were used to estimate $\mu_{0}$, the next $\hat{n}_{1}$ observations were used to estimate $\mu_{1}$, etc. Finally, $$\hat{\mu }_{d} = \left[ {\begin{array}{*{20}c} {\hat{\mu }_{0} } \\ \ldots \\ {\hat{\mu }_{G - 1} } \\ \end{array} } \right]$$ was used as both an input starting parameter vector and a prior by mclust for the contrast cluster means (corresponds to $\mu_{k}$ in [7]). A vector of $\left[ {\hat{n}_{1} /n, \ldots ,\hat{n}_{c} /n} \right]$ was used as starting proportions. All initial and prior variance parameters (see text regarding $\varSigma$ in [7]) were set to 0.06 since this is also the prior variance used by the APT software and should provide a good comparison of the methods. Both the means and the variances are updated in every iteration of the EM-algorithm, ending with the maximum likelihood estimates of the parameters. The models assume a normal distribution for each cluster with different means. For the models ‘E’ (equal) and ‘V’ (varying), the variances are either equal or different, respectively, for all clusters.

## References

[1] Bourke PM, Voorrips RE, Visser RGF, Maliepaard C (2018). Tools for genetic studies in experimental populations of polyploids. Front Plant Sci.

[2] Song C, Liu S, Xiao J, He W, Zhou Y, Qin Q (2012). Polyploid organisms. Sci China Life Sci.

[3] Chester-Jones I, Ingelton PM, Phillips JG (1987). Fundamentals of comparative vertebrate endocrinology.

[4] Piferrer F, Beaumont A, Falguière JC, Flajšhans M, Haffray P, Colombo L (2009). Polyploid fish and shellfish: production, biology and applications to aquaculture for performance improvement and genetic containment. Aquaculture.

[5] Fisher T. GeneTitan multi-channel (MC) instrument. 2018. https://www.thermofisher.com/no/en/home/life-science/microarray-analysis/microarray-analysis-instruments-software-services/microarray-analysis-instruments/genetitan-multi-channel-instrument.html. Accessed 13 Mar 2020.

[6] Fisher T. Affymetrix power tools. 2018. https://www.thermofisher.com/no/en/home/life-science/microarray-analysis/microarray-analysis-partners-programs/affymetrix-developers-network/affymetrix-power-tools.html. Accessed 13 Mar 2020.

[7] Scrucca L, Fop M, Murphy TB, Raftery AE (2016). mclust 5: clustering, classification and density estimation using Gaussian finite mixture models. R J.

[8] Biernacki C, Celeux G, Govaert G (2000). Assessing a mixture model for clustering with the integrated completed likelihood. IEEE Trans Pattern Anal.

[9] Fisher T. Axiom genotyping solution data analysis guide. https://assets.thermofisher.com/TFS-Assets/LSG/manuals/axiom_genotyping_solution_analysis_guide.pdf.

[10] Brooker RJ, Widmaier EP, Graham LE, Stiling PD (2011). Biology.

[11] Thorgaard GH, Allendorf FW, Knudsen KL (1983). Gene-centromere mapping in Rainbow trout: high interference over long map distances. Genetics.

[12] ICSASG_v2 NCBI Assembly: International cooperation to sequence the Atlantic salmon genome. 2015. https://www.ncbi.nlm.nih.gov/assembly/GCF_000233375.1/#/def_asm_Primary_Assembly. Accessed 13 Mar 2020.

[13] Lien S, Koop BF, Sandve SR, Miller JR, Kent MP, Nome T (2016). The Atlantic salmon genome provides insights into rediploidization. Nature.

[14] Lien S, Gidskehaug L, Moen T, Hayes BJ, Berg PR, Davidson WS (2011). A dense SNP-based linkage map for Atlantic salmon (*Salmo salar*) reveals extended chromosome homeologies and striking differences in sex-specific recombination patterns. BMC Genomics.

[15] O’Flynn FM, McGeachy SA, Friars GW, Benfey TJ, Bailey JK (1997). Comparisons of cultured triploid and diploid Atlantic salmon (*Salmo salar* L.). ICES J Mar Sci.

[16] Pereira GS, Garcia AAF, Margarido GRA (2018). A fully automated pipeline for quantitative genotype calling from next generation sequencing data in autopolyploids. BMC Bioinformatics.

[17] Clark LV, Lipka AE, Sacks EJ (2019). polyRAD: genotype calling with uncertainty from sequencing data in polyploids and diploids. G3 (Bethesda).

[18] Voorrips RE, Gort G. fitPoly: genotype calling for bi-allelic marker assays. Version 3.0.0. 2018. https://github.com/cran/fitPoly. Accessed 13 Mar 2020.

[19] Voorrips RE, Gort G, Vosman B (2011). Genotype calling in tetraploid species from bi-allelic marker data using mixture models. BMC Bioinformatics.

[20] Serang O, Mollinari M, Garcia AA (2012). Efficient exact maximum a posteriori computation for bayesian SNP genotyping in polyploids. PLoS One.

[21] Zych K, Gort G, Maliepaard CA, Jansen RC, Voorrips RE (2019). FitTetra 2.0—improved genotype calling for tetraploids with multiple population and parental data support. BMC Bioinformatics.

[22] Miclaus K, Wolfinger R, Vega S, Chierici M, Furlanello C, Lambert C (2010). Batch effects in the BRLMM genotype calling algorithm influence GWAS results for the Affymetrix 500 K array. Pharmacogenomics J.

[23] Clayton DG, Walker NM, Smyth DJ, Pask R, Cooper JD, Maier LM (2005). Population structure, differential bias and genomic control in a large-scale, case–control association study. Nat Genet.

[24] Hong H, Su Z, Ge W, Shi L, Perkins R, Fang H (2008). Assessing batch effects of genotype calling algorithm BRLMM for the Affymetrix GeneChip Human Mapping 500 K array set using 270 HapMap samples. BMC Bioinformatics.

[25] McLachlan GJ, Peel D (2000). Finite mixture models.

[26] Thermo Fisher. BRLMM-P: a genotype calling method for the SNP 5.0 array. 2007. https://www.google.fr/url?sa=t&rct=j&q=&esrc=s&source=web&cd=2&ved=2ahUKEwiqjLeUlannAhWQmBQKHSlbCvsQFjABegQIChAF&url=http%3A%2F%2Ftools.thermofisher.com%2Fcontent%2Fsfs%2Fbrochures%2Fbrlmmp_whitepaper.pdf&usg=AOvVaw1F_EnjOHCE1r6JCcGbFvbR. Accessed 13 Mar 2020.

[27] Hayes BJ (2011). Efficient parentage assignment and pedigree reconstruction with dense single nucleotide polymorphism data. J Dairy Sci.

[28] Grashei KE, Odegard J, Meuwissen THE (2018). Using genomic relationship likelihood for parentage assignment. Genet Sel Evol.

[29] Strucken EM, Lee SH, Lee HK, Song KD, Gibson JP, Gondro C (2016). How many markers are enough? Factors influencing parentage testing in different livestock populations. J Anim Breed Genet.

[30] Miller PA, Elliott NG, Vaillancourt RE, Koutoulis A, Henshall JM (2016). Assignment of parentage in triploid species using microsatellite markers with null alleles, an example from Pacific oysters (*Crassostrea gigas*). Aquacult Res.

[31] Eisbrenner WD, Botwright N, Cook M, Davidson EA, Dominik S, Elliott NG (2014). Evidence for multiple sex-determining loci in Tasmanian Atlantic salmon (*Salmo salar*). Heredity.

[32] Kijas J, McWilliam S, Naval Sanchez M, Kube P, King H, Evans B (2018). Evolution of sex determination loci in Atlantic salmon. Sci Rep.

[33] Chourrout D, Chevassus B, Krieg F, Happe A, Burger G, Renard P (1986). Production of second generation triploid and tetraploid rainbow trout by mating tetraploid males and diploid females—potential of tetraploid fish. Theor Appl Genet.

[34] Liu S (2010). Distant hybridization leads to different ploidy fishes. Sci China Life Sci.

